# Effects of Maternal Clofibrate Supplementation During Gestation and Lactation on Intestinal Fatty Acid Oxidation of Suckling Piglets

**DOI:** 10.3390/ijms26178691

**Published:** 2025-09-06

**Authors:** Brandon Pike, Jinan Zhao, Julie A. Hicks, Feng Wang, Paige Meisner, Lin Yang, Hsiao-Ching Liu, Jack Odle, Xi Lin

**Affiliations:** Laboratory of Developmental Nutrition, Department of Animal Sciences, North Carolina State University, Raleigh, NC 27695, USA; bepike2@ncsu.edu (B.P.); jnzhao@zinpro.com (J.Z.); jahicks3@ncsu.edu (J.A.H.); fwang22@ncsu.edu (F.W.); pmeisner@umd.edu (P.M.); lyang32@ncsu.edu (L.Y.); hc_liu@ncsu.edu (H.-C.L.); jodle@ncsu.edu (J.O.)

**Keywords:** clofibrate, fatty acid (FA), intestinal mucosa, maternal, suckling piglets

## Abstract

To accelerate maturation of intestinal function and promote growth and development, the effect of maternal clofibrate on intestinal fatty acid (FA) metabolism was investigated in suckling piglets. Twenty-seven pregnant sows were fed either 0, 0.25, or 0.5% clofibrate, a peroxisome proliferator-activated receptor α (PPARα) agonist, during late gestation and early lactation. [1-^14^C]-Oleic acid metabolism was measured in vitro in intestinal mucosa of piglets with/without L-carnitine and/or malonate. Clofibrate increased oleic acid metabolism on d1, and the increase was higher from 0.5% than 0.25% of maternal clofibrate (*p* < 0.005). Flux to CO_2_ increased with age, while flux to acid-soluble products (ASP) remained constant after d1. Flux to esterified products (ESP) increased on d7, but the increase was dampened by clofibrate (*p* < 0.0001). Carnitine increased flux to CO_2,_ and malonate decreased it (*p* < 0.0001), but neither affected ASP or ESP. Intestinal non-esterified FA and triglyceride levels decreased linearly, and carnitine palmitoyl-transferase (CPT) activity increased quadratically with age. Clofibrate increased acyl-CoA oxidase (*ACOX*) abundance but decreased the ratio of *CPT1A* and *CPT1B* on d1. Postnatal age increased FA binding protein 2 (*FABP2*) but decreased *PPARα*. In conclusion, maternal supplementation of clofibrate promotes intestinal energy generation from fat oxidation in postnatal piglets, but the stimulation is influenced by age, in which ACOX, FABP2, and CPT1 might play modulatory roles.

## 1. Introduction

The permeability of porcine placenta to fatty acids (FAs) is highly restricted, and consequentially, the primary energy substrate for the developing fetus is glucose. Ketone bodies may be used as fuel and lipogenic substrates by the fetus when the mother is under fasting conditions, using FA for ketogenesis [1]. Thus, fetal ketone bodies play a crucial role in transition from glucose-based metabolism to fat-based metabolism. Fetal ketones also may influence enterocyte differentiation and help to maintain stem cells in the intestine. However, milk lipids become the principal substrate for oxidative metabolism after birth. More than 55% of the dietary energy for neonates is milk fat [2], depending on species, suggesting that efficient postnatal oxidation of FA is critical to support growth and development. Unlike human infants, newborn piglets and rats [3] have very limited fat stores. Energy is derived primarily from absorbed FA from the milk lipids, implying that the intestine plays a critical role for neonates to acquire energy efficiently after birth. Increasing intestinal adaptation to the source of energy will improve the ability of piglets to absorb and oxidize fat, contributing to improved survivability.

Lipids from milk are absorbed primarily from the small intestinal lumen into the lymph system via the intestinal epithelial cells. This process undergoes significant development throughout life, with key changes happening during fetal development and neonatal growth. The changes during development affect the efficiency of lipid uptake depending on species and energy needs at different stages. Fetal and neonatal pigs, like immature infants, are enriched in fetal-type cells in the developing intestine [4]. The fetal-type cells must rapidly mature after birth into fully functional adult enterocytes. This transformation is crucial for their survival, development, and growth, as they have limited endogenous energy stores. To attain adult absorptive and secretory functions, therefore, the energy requirement of the intestine is very high [5], providing the needs for synthesis of digestive enzymes and the rapid renewal of enterocytes.

The role of peroxisome proliferator-activated receptor alpha (PPARα) in FA oxidation in the liver has been well established, as the liver is the primary location of FA metabolic and ketogenic pathways to generate energy for use by other tissues. Because the intestine utilizes lipids for energy itself [6], we aimed to evaluate the regulatory function of PPARα in the intestine. Most studies examining the effects of PPARα activation on intestinal lipid metabolism were performed in rodents [7,8,9,10]. Specifically, the expression of genes associated with PPARα activation by clofibrate, its pharmaceutical agonist, was determined in the developing small intestine in rats [11]. The results revealed that the PPARα target genes, such as acyl-CoA oxidase 1 *(ACOX 1*) and FA-binding proteins (*FABPs*), might be coordinately regulated during postnatal development. Moreover, increased FA oxidation was observed also in enterocytes treated with the PPARα agonist bezafibrate [8]. As a non-peroxisome-proliferating species [12], however, the kinetic responses of intestinal FA oxidation of pigs to PPARα activation have not been explored during the neonatal-suckling period. Optimizing the metabolism of FA in the small intestine is beneficial for the survival and development of newborn pigs, because high neonatal piglet mortality has remained a major economic and animal welfare problem in the swine industry worldwide for decades.

The high mortality observed in neonatal swine is associated with low energy intake and utilization after birth [13]. Increasing milk lipid utilization can improve growth and development, subsequently reducing mortality. Results from our previous studies showed that supplementation of clofibrate to neonatal piglets promoted hepatic and extrahepatic PPARα-dependent gene expression and increased FA oxidation [14,15]. Specifically, we found that maternal clofibrate could be transferred to the fetus across the placenta membrane and increase FA oxidation at birth. However, the stimulated FA oxidation was diminished with time [16], suggesting that sustained PPARα activation is required to maintain the high FA oxidation rate after birth. In addition, lactation (milk) transfer of clofibrate was observed in suckling rats, resulting in increased hepatic and renal cytochrome *P450 4A* mRNA levels [17,18]. Because clofibrate can be transferred from mother to fetus and newborn via placenta and milk, we hypothesized that supplementation of clofibrate to sows during late gestation and early lactation would stimulate utilization of milk FA in the rapidly developing intestine of their offspring via activation of PPARα. To test our hypothesis, the capacity of energy generation from FAs was evaluated over time in the intestine of suckling piglets from sows treated with or without clofibrate during late gestation and early lactation.

## 2. Results

### 2.1. FA Oxidation

#### 2.1.1. Effects of Maternal Supplementation of Clofibrate on FA Metabolism in the Intestinal Mucosa of Piglets During the Neonatal-Suckling Period

Significant interactions between maternal supplementation of clofibrate and postnatal age were observed for the ^14^C accumulation in CO_2_ (*p* < 0.005), acid soluble products (ASP; *p* < 0.05), and esterified products (ESP; *p* < 0.0001) as well as the total oxidation (*p* < 0.05) and total metabolism (*p* < 0.0001) from oleic acid (Table 1).

The ^14^CO_2_ accumulation was stimulated linearly with the dose of maternal clofibrate on d1 (*p* < 0.0005), but the stimulation was diminished after d7, depending on the dose. The accumulation rate measured on d1 was 1.1 and 1.6-fold greater in piglets from sows fed 0.25 and 0.5% clofibrate than in controls. The difference was not detectable on d7, while the accumulation was 60% higher on d19 from 0.25% and 44% and 72% higher on d14 and d19 from 0.5% clofibrate-treated sows than from the controls (*p* < 0.05).

The ^14^C accumulation in ASP on d1 was 1.3 and 1.7-fold higher in pigs from sows with 0.25 and 0.5% clofibrate than from the controls (*p* < 0.05), but maternal supplementation had no effects on the ^14^C accumulation in ASP in pigs after d1. The ^14^C accumulation in ASP was higher from d1 than all other ages (*p* < 0.0001), and no differences were observed between all other ages.

The ^14^C accumulation in ESP in piglets from control sows increased from d1 to 7 but decreased greatly after d7 (*p* < 0.005). The accumulation was on average 61% and 81% lower from d14 and d19 than from d1 and d7. No difference was detected between d14 and d19. Maternal clofibrate had no impact on the accumulation in ESP on d1, 14, and 19, but decreased the accumulation in ESP on d7 (*p* < 0.0001). The decrease was greater in pigs from sows fed 0.5% than 0.25% clofibrate.

The ^14^C accumulation in total oxidation (CO_2_ + ASP) was 37% and 79% higher in piglets on d1 from sows with 0.25% and 0.5% clofibrate than from the controls (*p* < 0.05), but supplementation had no effect on the ^14^C accumulation after d1. The ^14^C accumulation in CO_2_ + ASP was higher from d1 than that measured from other ages (*p* < 0.0001), but no differences were observed between all other ages.

The ^14^C accumulation in the total metabolism (CO_2_ + ASP + ESP) in piglets from control sows increased from d1 to d7 (*p* < 0.005) but had no difference on d14 and d19. The accumulation was 3.1 and 4.6-fold higher on average from d1 and 7 than from d14 and d19 (*p* < 0.0001). Maternal supplementation of 0.5% clofibrate increased the total metabolism on day 1 (*p* < 0.005) but decreased the accumulation in ESP on d7. The decrease was greater in sows fed 0.5% than 0.25% clofibrate.

#### 2.1.2. Effects of Maternal Supplementation of Clofibrate on Distribution (%) of CO_2_, ASP and ESP in Total FA Oxidation and Metabolism in the Intestinal Mucosa of Piglets During the Neonatal-Suckling Period

The % CO_2_ in total FA oxidation increased, and the % of ASP in Total FA oxidation decreased linearly with maternal clofibrate dose (*p* < 0.05) and postnatal age (*p* < 0.0001). No interactions (*p* > 0.1) were detected between maternal clofibrate and postnatal age (Table 2).

The % of CO_2_, ASP, and ESP in total FA metabolites had significant (*p* < 0.005) interactions (Table 2) between the maternal clofibrate and age. The % of CO_2_ in total metabolites increased with age from d1 to d14 (*p* < 0.0001), but the increase was greater in pigs from sows fed 0.25% clofibrate on d7. The % CO_2_ in pigs on d14 and 19 from sows fed 0.5% clofibrate were higher than that from control sows (*p* < 0.05). The % of CO_2_ in pigs from sows fed 0.25% clofibrate was lower at d 19 compared to d14 (*p*< 0.005), but the % measured in pigs from sows fed 0 or 0.5% clofibrate showed no difference. The % of ASP in total metabolites measured in piglets from control sows was decreased from d1 to d7 and then increased from d7 to d19 (*p* < 0.0001). Maternal clofibrate increased the % of ASP, but the increase varied with age and clofibrate dose. The % was higher in piglets on d1 and 14 from sows fed 0.25% clofibrate than control sows, and on d7 and d14 from sows fed 0.5% clofibrate than the controls (*p* < 0.05). The % of ESP in total metabolites in pigs from control sows increased from d1 to d7 but decreased after d7 (*p* < 0.005). As opposed to the % of ASP, maternal supplementation of clofibrate decreased the % of ESP, and the decrease varied with the age and dose of maternal clofibrate supplementation. The decrease in % of ESP from 0.5% of clofibrate was similar to 0.25% of clofibrate on d1 and d7 but was greater than 0.25% of clofibrate on d14 and 19 (*p* < 0.05).

There was no interaction between maternal clofibrate and age on the ratio of CO_2_ and ASP (C/A). Maternal clofibrate also had no impact on the ratio, but the ratio increased with age. On average, the ratio increased by 2.75-fold after d7 compared to d1 (*p* < 0.0001). The ratio of oxidized and metabolized products (O/M) in piglets from the control sows decreased by 34% from d1 to d7 but increased by 106% from d7 to d14 and by 37% from d14 to d19 (*p* < 0.001). Maternal clofibrate had no impact on the ratio at d1 and d7 but increased greatly after d7. The ratio in piglets from clofibrate-fed sows was on average 13% and 39% higher than control sows (*p* < 0.005).

#### 2.1.3. Effects of Carnitine and Malonate on FA Metabolism in the Intestinal Mucosa of Piglets During the Neonatal-Suckling Period

**Figure 1:**
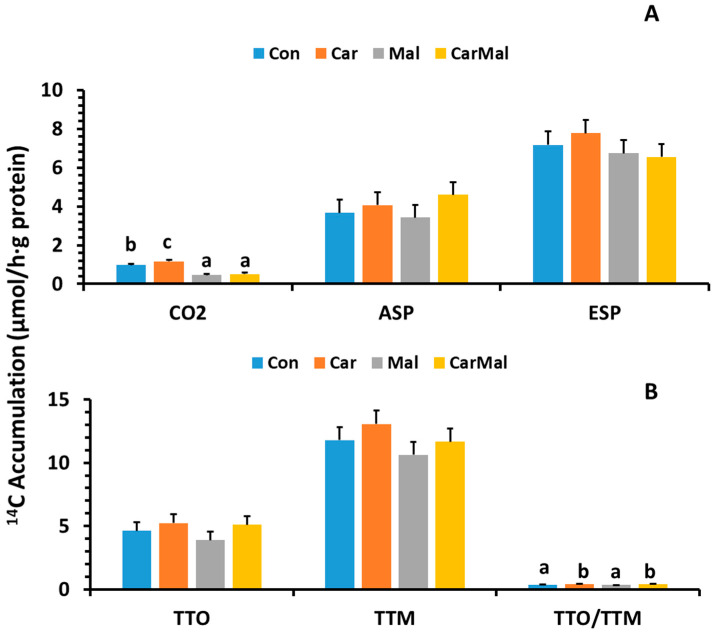
Effects of carnitine and malonate on intestinal oleic acid metabolic products (**A**) and total intestinal oleic acid oxidation and metabolism (**B**) in suckling piglets. Oleic acid oxidative metabolism was measured with or without carnitine (1 mM) and/or malonate (5 mM) in intestinal mucosa of pigs from sows with or without clofibrate (Con, control; Car, carnitine; Mal, malonate; CarMal, carnitine + malonate). Columns represent oxidative products (µmol/g protein. h) and are least square means ± standard error means (CO_2_; ASP, acid soluble product; ESP, esterified products; TTO, total oxidation product; TTM, total metabolic products; TTO/TTM, the ratio of TTO and TTM). ^abc^ Columns with differing letters denote significant differences (*p* < 0.05).

No interaction was detected between maternal clofibrate (*p* = 0.9) and the tissue treatment with carnitine or malonate for CO_2_ production. The main effects are presented in Figure 1A. The supplementation of carnitine increased ^14^C accumulation in CO_2_ by 18% compared to control (*p* < 0.05). Supplementation of malonate decreased ^14^C accumulation in CO_2_ by 61% compared to control (*p* < 0.0001). No improvement was observed after adding carnitine to the treatment with malonate. Supplementation of carnitine and/or malonate in the mucosa incubation had no impact on the accumulation in ASP (*p* > 0.1). No interaction was detected between maternal clofibrate (*p* > 0.1) and the tissue treatment with carnitine or malonate. The tissues from all pigs treated with carnitine and malonate had no impact on the ESP production (*p* > 0.1).

Total oxidized products (CO_2_ + ASP) and the total metabolites (CO_2_ + ASP + ESP) were not affected by supplementation of carnitine and/or malonate (*p* > 0.1). No interaction was detected between maternal clofibrate (age) and the tissue treatment with carnitine or malonate. As the main effect (Figure 1B), addition of carnitine increased the ratio of O/M regardless of malonate treatment (*p* < 0.0001), but no interaction was detected between maternal clofibrate (age) and the tissue treatment with carnitine or malonate.

#### 2.1.4. Effects of Carnitine and Malonate on Distribution (%) of CO_2_, ASP, and ESP in Total FA Oxidation and Metabolism in the Intestinal Mucosa of Piglets During the Neonatal-Suckling Period

There was no interaction between maternal clofibrate treatments and the treatments with carnitine and/or malonate (*p* > 0.05). However, significant interactions were detected between adding carnitine and/or malonate and postnatal age for % of CO_2_ and ASP (*p* < 0.01) in total oxidation and the C/A (*p* < 0.0001), as well as the % of CO_2_ (*p* < 0.0001) and ASP (*p* < 0.01) in the total metabolism. The interaction for ESP % in the total metabolism and the O/M also tended to be significant (*p* = 0.052).

**Figure 2:**
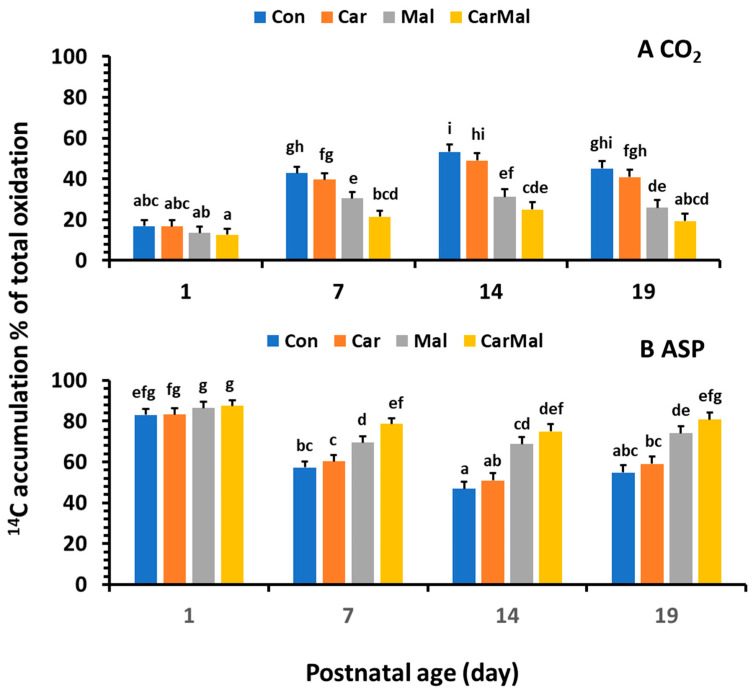
Effects of carnitine and malonate on the distribution of intestinal oleic acid oxidation in piglets during the suckling period. Oleic acid oxidation was measured with or without carnitine (1 mM) or/and malonate (5 mM) in intestinal mucosa of pigs from sows with and without clofibrate (Con, control; Car, carnitine; Mal, malonate; CarMal, carnitine + malonate). Columns represent % of total oxidative products and are least square means ± standard error means for CO_2_ (**A**) and ASP, acid soluble product (**B**). ^abcdefghi^ Columns lacking a common letter are different (*p* < 0.05).

Addition of carnitine in the incubation medium had no influence on the % of CO_2_ and ASP in the total oxidized products (*p* > 0.1), but addition of malonate reduced the % of CO_2_ (Figure 2A) and increased the % of ASP (Figure 2B) in all ages (*p* < 0.05). The decrease was greater from the addition of carnitine + malonate than from malonate when compared to the control. The ratio of CO_2_/ASP in the control group increased quadratically with age, but the increase was reduced by the addition of carnitine. The addition of malonate inhibited the ratio increase with age and kept the ratio with no difference from the d1.

**Figure 3:**
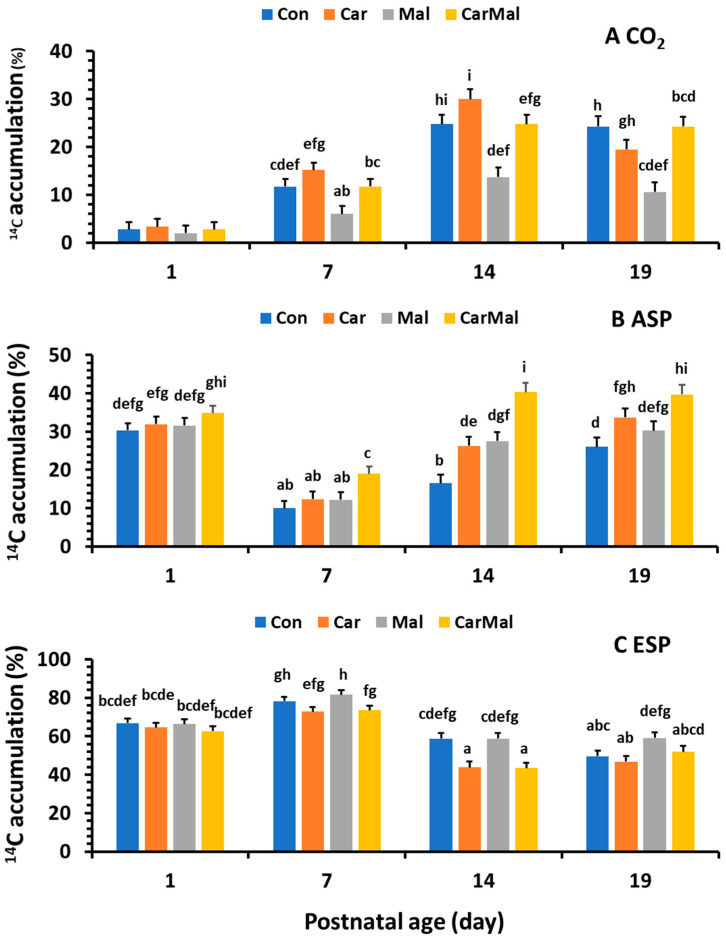
Effects of carnitine and malonate on the distribution of intestinal oleic acid metabolism in piglets during the suckling period. Oleic acid oxidation was measured with or without carnitine (1 mM) or/and malonate (5 mM) in intestinal mucosa of pigs from sows with and without clofibrate (Con, control; Car, carnitine; Mal, malonate; CarMal, carnitine + malonate). Columns represent % of total metabolic products and are least square means ± standard error means for CO_2_ (**A**); ASP, acid soluble product (**B**); and ESP, esterified products (**C**). ^abcdefghi^ Columns lacking a common letter are different (*p* < 0.05).

A similar pattern of % of CO_2_ (Figure 3A) as in oxidation was observed in the control group. However, the % of ASP (Figure 3B) significantly decreased after d1, and the decrease was greater from d7 than from d14 and 19. Addition of carnitine or/and malonate increased % of ESP (Figure 3C), and the increase was greater from carnitine + malonate than from carnitine or malonate only. No difference was observed between d14 and d19. The % of ESP increased on d7 and decreased after d7. The increase was reduced by the addition of carnitine and increased by the addition of malonate. No impacts were detected after d7. The ratio of oxidation and metabolism followed the same pattern as observed in % ASP.

### 2.2. Non-Esterified Fatty Acid (NEFA) and Triglyceride (TG) Concentrations

**Figure 4:**
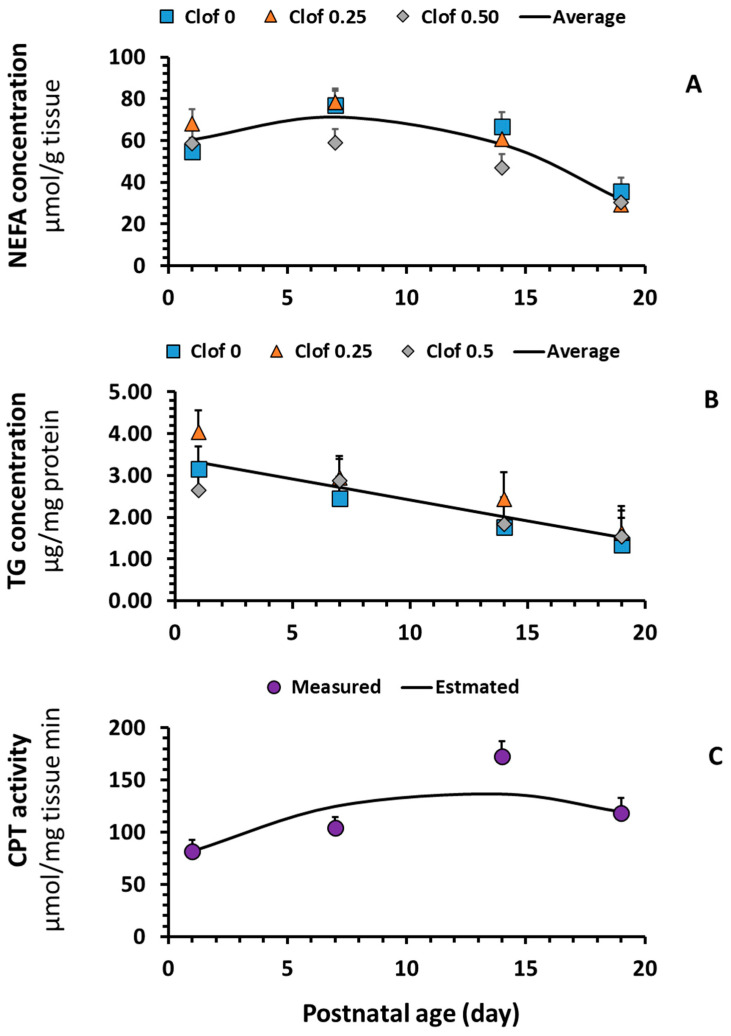
Effect of postnatal age on intestinal NEFA, TG, and CPT activity in suckling piglets. NEFA (**A**) and TG (**B**) were determined using the commercial kits from MyBioSource Inc (MBS9719080 and MBS2556986; San Diego, CA, USA). The type and color of the markers represented data from maternal treatments (Clof 0, control; Clof 0.25 clofibrate 0.25; Clof 0.5 clofibrate 0.5), and the curve was from regression analysis with data from all tissue treatments (R^2^ = 0.2 and *p* < 0.0001) for NEFA and (R^2^ = 0.17 and *p* < 0.001) for TG. CPT activity (**C**); (R^2^ = 0.1 and *p* < 0.05).

Maternal clofibrate supplementation had no impact on NEFA and TG concentration in the intestinal mucosa (*p* > 0.1). The average NEFA and TG levels in the tissue were 52.94 ± 5.47 (µmol/g) and 2.39 ± 0.30 (μg/mg protein), respectively. However, both NEFA (Figure 4A) and TG (Figure 4B) varied greatly with postnatal age, in which a significant linear decrease with postnatal age was observed in the TG concentration and a quadratic response to the postnatal age was observed in NEFA concentration (*p* < 0.005). No interaction (*p* > 0.05) was detected between maternal clofibrate and postnatal age.

### 2.3. Carnitine Palmitoyltransferase (CPT) Enzyme Activity

Maternal supplementation of clofibrate had no significant impact on the activities of CPT1, CPT2, and Total (CPT1 + CPT2), except for the trends test using orthogonal polynomial coefficients that showed the CPT1 activity tended to increase linearly with maternal clofibrate dose (*p* = 0.067). Postnatal age had a greater impact on CPT2 activity and the total activity of CPT (*p* < 0.001), and the impact followed a quadratic pattern (*p* < 0.01). The highest activity was observed in piglets at 14 days of age (Figure 4C).

### 2.4. Gene Expression (qPCR)

Maternal supplementation of clofibrate had no impact on the expression of genes examined in the intestinal mucosa of pigs (Table 3). However, postnatal age had an impact on the expression of *CPT1A* (*p* < 0.05), *FABP2* (*p* < 0.001), *PPARα* (*p* < 0.05), and Retinoid X receptor alpha (*RXRα*; *p* < 0.005). The expression of *FABP2* was, on average, 90% higher in pigs at d14 and 19 than at d1 and d7, while the expression of *CPT1A* was 53% lower on d14 than on the average of d1, d7, and d19. *PPARα* and *RXRα* were on average 50% and 74% lower in pigs at d19 than at d1 and d7. In addition, the interaction between maternal clofibrate and postnatal age was significant for *ACOX* expression (*p* < 0.05) and tended to be significant for the ratio of *CPT1A* and *CPT1B (p =* 0.087), in which maternal supplementation of clofibrate increased ACOX expression but decreased the ratio of *CPT1A* and *CPT1B* on d1.

## 3. Discussion

### 3.1. The Effect of Maternal Clofibrate on Intestinal FA Metabolism in Suckling Piglets

Development and growth of the intestine is rapid after birth, and increasing FA oxidation could be important for the intestine to meet the energy requirement and promote the fetal-type enterocyte maturation because milk fat is the primary energy source at birth. In this study, we evaluated the effects of PPARα activation on intestinal FA metabolism in piglets by feeding the sows clofibrate (a PPARα agonist) during late gestation and early lactation. Maintaining a high fatty acid oxidation rate during the first postnatal week is crucial because 90% of preweaning mortality occurs during this window. The effects of clofibrate varied with dose and piglet postnatal age. In general, the intestinal oxidation of oleate to CO_2_ increased linearly with the postnatal age. Maternal supplementation of clofibrate promoted the increase throughout the entire postnatal period. Consistent with the increase in CO_2_ production, mucosal concentrations of TG and NEFA decreased with age, congruent with their potential roles as energy substrates. Furthermore, stimulation of CO_2_ production was impacted by clofibrate doses and by postnatal age, despite clofibrate being undetectable in the milk. Similar results were not observed in liver metabolism with the same treatments after d1 [14]. Given the detection limit of measuring clofibrate, whether the variation was associated with a potentially very trace amount of clofibrate in the milk was not known. In addition, the variations in CO_2_ production were not in conjunction with an increase in CPT1 activity and gene expression of *PPARα* and *RXRα* in the present study, although a higher CPT2 activity was observed on d14. CPT1A and CPT1B are located on the mitochondrial membrane in most tissues. The pig has an atypical molecular structure of CPT1 in that the sensitivity of CPT1A and CPT1B to malonyl-CoA, the physiological inhibitor of CPT1, is different from other animals [19]. Specifically, pig CPT1A has a high sensitivity to malonyl-CoA inhibition, and CPT1B has a low sensitivity to malonyl-CoA inhibition [20]. Therefore, we examined the CPT1A and CPT1B gene expression and were unable to detect any changes in the intestine of piglets from sows with clofibrate supplementation in early lactation. However, we noticed that the ratio of *CPT1A/CPT1B* decreased with postnatal age. The interaction between maternal clofibrate and piglet age for the ratio tended to be significant, and the ratio was lower on d1 from clofibrate than control. The changes in *CPT1A* and *CPT1B* ratio might influence the sensitivity of CPT1 to malonyl-CoA inhibition, impacting fatty acid oxidation via regulating acetyl-CoA oxidation in the tricarboxylic acid cycle (TCA). Therefore, the relative decrease in highly malonyl-CoA-sensitive CPT1A might be associated with the CO_2_ increase. In addition, interaction between maternal clofibrate and postnatal age for *ACOX1* expression was detected, with a higher expression on d1. Also, *FABP2* expression increased with age, indeed, suggesting that ACOX1 and FABP2 play a role in intestinal FA oxidation as observed in rats [11]. Furthermore, it is possible that genes associated with the TCA that were not examined in this study contributed to the increase in CO_2_ production. CO_2_ production is indicative of increased ATP production through the TCA, and it has been reported that intermediates of the TCA were altered with clofibrate treatment [21].

Compared to CO_2_, ASP production had completely different responses. The dose-dependent stimulation of oleate oxidation to ASP by maternal clofibrate was detected only on d1. Combining CO_2_ and ASP, the effect of maternal clofibrate on FA oxidation followed the same pattern as observed in ASP production. This result was consistent with that observed in the liver of piglets receiving clofibrate from maternal supplementation, demonstrating clofibrate transferred from sows to fetal piglets via the placenta. After d1, ASP production decreased dramatically, and the reduced ASP production was not affected by maternal clofibrate supplementation or by further advancement in postnatal age. The lack of response to maternal clofibrate could be due to a low mammary transfer efficiency or dose of clofibrate supplementation, because the dose used in the previous study with gestation sows was at least 60% [16] higher than the dose we fed to the sows in this study. In addition, ketogenic capability was observed in the small intestine of rats [22] and can be induced by a ketogenic diet [23], but the expression of ketogenic rate-limiting enzyme mitochondrial 3-hydroxy-3-methylglutaryl-CoA synthase (HMGCS) was not detectable in the small intestine of neonatal pigs [24]. Butyrate was converted into ketone bodies observed in colonocytes with increasing concentrations of [1-^14^C] butyrate [25], but gene expression was almost not detected in the duodenum, jejunum, and ileum until 8 weeks of age. Therefore, the reduced ASP production appeared to be associated with the inherent defect in ketogenic ability in the intestine of neonatal piglets. In addition, *PPARα* and *RXRα* significantly decreased after d7. The reduced ASP production appeared to be associated with the lack of ketogenic ability and gene expression, while the high ASP production on d1 could be related to the high-level TG and NEFA, as well as the accumulation of the absorbed soluble carnitine esters after birth.

A previous study with hepatocytes isolated from suckling newborn and 15 d old pigs showed that neonatal piglets have a huge rate of hepatic FA esterification [26]. The rate of FA esterification was associated with the rapid increase in fat stores after birth [27]. Indeed, the hepatic esterification increased greatly with the postnatal age [14]. A similar increase in ESP production on d2 was also observed previously with pig intestinal cells [28]. It is very interesting, however, that the intestinal esterification rate increased after birth and decreased greatly after 7 days, and that maternal clofibrate had a great impact on the increase in FA esterification on d7. The high ESP measured in pigs from control sows was greatly reduced compared to pigs from sows with clofibrate. The reduction was much greater in sows fed high clofibrate than low clofibrate, suggesting that maternal clofibrate could affect the ESP after birth, and the degree of effect depended on the dosage of maternal clofibrate. However, no clofibrate effect was detected when the ESP decreased quickly with age after d7. These changes were consistent with the decrease in *PPARα* and *RXRα* expression and the increase in *FABP2* expression with age. The results from this study highlighted the high esterification capacity for FA in the newborn pig intestine, which may preserve this major colostrum FA for delivery to other tissues. Nutritional control of intestinal FA esterification, such as dietary fat intake, has been described in the literature [29]. Milk fat is usually high after farrowing and in the first several days after farrowing, depending on species. Sow milk fat peaked around d3 and then decreased as lactation progressed [30]. We were interested in the potential influence of the decreased ESP on FA oxidation. The decrease in esterification on d7 and after d7 clearly illustrates that it has a limited impact on overall oxidation. Combining the oxidative products and ESP, the total metabolites were increased at d1 primarily due to an increase in oxidation induced by clofibrate, while the total metabolism was decreased at d7 primarily due to a decrease in esterification.

Although the stimulatory effect of clofibrate on FA oxidation had a significant interaction with postnatal age, the interaction did not affect oxidative product distributions between CO_2_ and ASP. In general, the CO_2_ increased with clofibrate dose and postnatal age, while the ASP decreased with clofibrate and postnatal age. Both linear and quadratic responses were detected. However, the distributions of CO_2_, ASP, and ESP in total metabolism were impacted significantly by maternal clofibrate and postnatal age. In general, the % of oxidative products on average increased, and the % of ESP decreased in piglets from sows receiving clofibrate during late gestation and early lactation. The effect of maternal clofibrate on the distribution of metabolic products appeared to be different from that in the liver [14], implying the tissue specificity in which the absorption of dietary FA and converting them into TG for transport to other tissues is the primary pathway, but not for producing ketone bodies and FA synthesis. The limited FA synthetic activity and rapid reduction in FA esterification are also the key factors in altering the metabolic allocations.

### 3.2. The Effect of Providing Carnitine and Inhibiting TCA Activity on Intestinal FA Metabolism in Suckling Pigs

Providing carnitine increased CO_2_ production and the oxidative proportion of the total metabolism, suggesting that the stimulatory role of carnitine might be limited to the entry of FA into mitochondria. This was confirmed by inhibiting TCA cycle activity via the addition of malonate, an inhibitor of succinate dehydrogenase. As the product of β-oxidation, acetyl-CoA can be metabolized completely via the TCA cycle or be converted to acetate, acetyl-carnitine, HMG-CoA for ketogenesis, or malonyl-CoA for FA synthesis, which comprises most of the ASP. We expected that ASP would increase when carnitine was added into the system, and both ASP and ESP would increase when the TCA cycle was inhibited. However, no influences on ASP and ESP were detected by the addition of carnitine and/or malonate. Available evidence in the literature showed that lipogenesis in pigs is extremely low in tissues other than adipose [31]. Consistent with this observation, the activities of enzymes related to lipogenesis are also very low in the intestine [32]. In addition to lipogenesis, the gene of the key enzyme HMGCS for the ketogenic pathway is undetectable in the small intestine of neonatal pigs [24]. The lack of lipogenic and ketogenic activities appeared to be associated with the lower response of ASP to carnitine and malonate. Although the status of carnitine acetyltransferase and acetyl-hydrolase in pig intestine was not evaluated in this study, and is not available in the literature, our results illustrated and supported that the energy generation pathway is the main flux within intestinal FA metabolism. Energy generated from FA oxidation is the primary fate in intestinal mucosal FA metabolism, such as the TG/FA absorption and FABP2 synthesis.

## 4. Materials and Methods

### 4.1. Animals and Treatments

The animal study was described previously [14]. Briefly, a total of 27 gestating sows with similar body weight (241.6 ± 9.9 kg) and parity (2.4 ± 0.9) in 3 blocks and 9 sows per block were divided into three groups and fed a standard commercial corn/soybean-meal diet (3265 kcal ME/kg) supplemented with three levels of clofibrate: 0% (Control), 0.25% (Clof 0.25), and 0.5% (Clof 0.5) of feed based on previous research with pigs [14,15], rats [33] and fish [34]. The treated sows received either clofibrate dissolved in 15 mL of ethanol or 15 mL of ethanol vehicle. The sows were fed twice daily, but the ethanol with or without clofibrate was given only in the morning via mixing with a small amount of feed. The clofibrate supplementation started one week pre-farrowing and ended one-week post-farrowing. The total litter size (including mummy and stillborn) was 16.8 ± 1.56 and was normalized among litters within 24 h by cross-fostering (within treatment group). All piglets were raised with their sows during the suckling period.

Three piglets on d1, d7, d14, and two piglets on d19 with average body weight were selected from each litter. The selected piglets were euthanized via American Veterinary Medical Association approved exsanguination while under anesthesia. Two intestinal segments (30 cm) were collected from the proximal and distal ends of the small intestine. The segments were rinsed with 0.9% NaCl solution and then opened lengthwise. Mucosa was obtained from the segments via a glass microscope slide. The mucosa from the first 30 cm was used for FA oxidation measurement, and the second 30 cm was frozen in liquid nitrogen for later analysis.

### 4.2. FA Metabolism Measurements

The collected mucosa was homogenized in a glass tissue grinder (7 mL, Vineland, NJ 08360, USA) in a buffer containing 220 mM mannitol, 70 mM sucrose, 2 mM N-2-hydroxyethylpiperazine-N-2-ethanesulfonic acid, 0.1mM EDTA with a ratio of 1:4 (*w*:*v*). FA oxidation was measured in a reaction buffer incubated with the fresh mucosa homogenates using [1-^14^C]-oleic acid as a substrate (1 mM; 0.25μCi/μmole). The measurements were performed in the presence or absence of L-carnitine (1 mM) with or without malonate (5 mM), an inhibitor of TCA, in a reaction buffer reported by Lin et al. [35]. The CO_2_, ASP, and ESP generated from the measurements and the homogenate protein were determined following the procedures described previously [14].

### 4.3. Non-Esterified Fatty Acid (NEFA) and Triglyceride (TG) Assays

TG and NEFA were determined in the frozen mucosa using commercial kits from MyBioSource Inc. (MBS9719080 and MBS2556986; San Diego, CA, USA).

### 4.4. Enzymatic Assay

Specific activity of carnitine palmitoyl transferase (CPT) was determined in the homogenate from the frozen mucosa samples with and without the addition of malonyl-CoA (10 mM) as described previously [36].

### 4.5. RNA Isolation and RT-qPCR

Total RNA was isolated from the mucosa using Tri reagent with 50 mg of the frozen samples following the manufacturer’s procedure. Quantification and quality control were performed using a Nanodrop spectrophotometer (ND-1000ThermoFisher, Wilmington, DE, USA) and 0.8% agarose gel electrophoresis post DNase treatment. cDNA synthesis was performed using Super Script III Reverse Transcriptase as described previously [15]. Primers for RT-qPCR (Supplemental Table S1) were created using BLAST Primer Designer (https://www.ncbi.nlm.nih.gov/tools/primer-blast/, accessed on 10 November 2016). The RT-PCR reaction and the CT values were normalized to a housekeeping gene (RPL9), and a plate normalizer was included on each plate to account for run differences. The relative changes in gene expression (normalized to newborn pigs) were calculated from the real-time PCR data using the 2^−ΔΔ^CT method, where ^ΔΔ^CT = (CT. Target − CT.GAPDH) age X − (CT. Target − CT.GAPDH) age 0 [37].

### 4.6. Chemicals

All chemicals for this study were sourced from Sigma-Aldrich, Inc. (St. Louis, MO 63103, USA), except for clofibrate, which came from Cayman Chemical (Ann Arbor, MI 48108, USA), Superscript from Thermo Fisher Scientific (Waltham, MA 02451, USA), and TurboDNase from Ambion. The ^14^C radiolabeled oleic acid and acetyl-CoA were purchased from American Radiolabeled Chemicals (St Louis, MO 63146, USA) and SYBR Green from BioRad (Hercules, CA 94547, USA).

### 4.7. Statistical Analysis

Data from enzymatic and RT-qPCR assays were analyzed according to a 3 (Control, Clof 0.25, and Clof 0.5) x 4 (d1, 7, 14, and 19) factorial randomized complete block design (blocked by sow (litter)). Data from in vitro FA oxidation was analyzed according to a split-plot design. The main plot was the 3 maternal dietary treatments on animals at 4 ages, and the sub-plot was the 4 treatments with a 2 (± carnitine) x 2 (± malonate) factorial design on the tissues. Analyses were performed using the General Linear Models (GLM) procedure of SAS (SAS software 9.4; Cary, NC, USA). Testing for trends (linear and quadratic) was also performed with the contrast statement using orthogonal polynomial coefficients. The least square means (Lsmeans) were calculated, and the interactions between clofibrate x postnatal age, and between (clofibrate x postnatal age) x treatments were tested. The data from main effects were reported only if the interaction was not significant. Data are presented as Lsmeans ± standard error means unless specified otherwise. Differences were reported as significant when *p* < 0.05 and as trends when 0.05 < *p* < 0.1.

## 5. Conclusions

Maternal supplementation of clofibrate during late gestation and early lactation improves intestinal energy generation via increasing β-oxidation activity, decreasing FA esterification, and promoting TG and NEFA utilization. This may be of great significance to newborn piglets with very limited adipose reserves at birth. However, the improvement varies with increasing postnatal age, in which the alterations of gene expression of *ACOX* and *FABP2α*, as well as the ratio of *CPT1A*/*CPT1B*, may also play regulatory roles. The in vitro addition of carnitine increases FA oxidation, but the increase is limited by the activity of the TCA cycle. Furthermore, the dose of clofibrate and the efficiency in the delivery of clofibrate via milk merit further investigation.

## Figures and Tables

**Table 1:** Effects of maternal clofibrate and postnatal age on oleic acid metabolism in suckling pigs.

Clofibrate		Con				Clof 0.25				Clof 0.50					*p*-Value	
Age	D1	D7	D14	D19	D1	D7	D14	D19	D1	D7	D14	D19	SEM	Clof	Age	Clof *Age
**Oxidation**					µmol/g protein. h											
CO_2_	0.31 ^a^	0.73 ^bcd^	0.80 ^bcd^	0.60 ^abc^	0.65 ^bc^	0.93 ^de^	0.82 ^bcde^	0.96 ^de^	0.82 ^cde^	0.54 ^ab^	1.15 ^e^	1.03 ^de^	0.11	0.0018 +	0.0020	0.0045 *
ASP	8.9 ^b^	1.07 ^a^	1.01 ^a^	1.35 ^a^	11.96 ^c^	1.14 ^a^	1.04 ^a^	1.86 ^a^	15.34 ^d^	0.75 ^a^	1.29 ^a^	1.57 ^a^	1.13	0.0990	0.0001 +^	0.0310
CO_2_ + ASP	9.22 ^b^	1.79 ^a^	1.81 ^a^	1.94 ^a^	12.61 ^c^	2.07 ^a^	1.86 ^a^	2.82 ^a^	16.16 ^c^	1.29 ^a^	2.43 ^a^	2.60 ^a^	1.15	0.0588	0.0001 +^	0.0246
**Metabolism**					µmol/g protein. h											
ESP	7.56 ^c^	22.83 ^e^	3.56 ^ab^	3.39 ^ab^	7.14 ^c^	16.17 ^d^	2.78 ^a^	3.53 ^ab^	7.41 ^c^	6.12 ^bc^	1.79 ^a^	2.37 ^a^	1.22	0.0001+	0.0001 +^	0.0001 *
CO_2_ + ASP + ESP	16.81 ^b^	24.57 ^d^	5.36 ^a^	5.44 ^a^	19.87 ^bc^	18.24 ^bc^	4.63 ^a^	6.34 ^a^	23.57 ^cd^	7.42 ^a^	4.25 ^a^	4.96 ^a^	1.81	0.0408	0.0001 +	0.0001

Data are least square means and pooled standard error means (SEM). ^abcde^ Data with different letter differ (*p* < 0.05). Con, Control sows, Clof 0.25, sows received 0.25% of dietary clofibrate and Clof 0.5, sows received 0.5% dietary clofibrate. ASP, acid soluble products and ESP, esterified products. * Indicating the difference between clofibrate and control from the analysis of contrast. + linear response. ^ quadratic response.

**Table 2:** Effects of maternal clofibrate and postnatal age on product distribution from oleic acid metabolism in suckling pigs.

		Con				Clof 0.25				Clof 0.50					*p*-Value	
	D1	D7	D14	D19	D1	D7	D14	D19	D1	D7	D14	D19	SEM	Clof	Age	Clof *
**Oxidation**																
CO_2_%	16.51	33.10	36.15	28.24	11.52	36.29	40.34	32.12	16.69	31.37	42.38	38.09	2.89	0.2410	0.0001 +^	0.1514
ASP%	83.49	66.90	63.85	71.76	88.48	63.71	59.66	67.88	83.31	68.63	57.62	61.91	2.89	0.2410	0.0001 +^	0.1514
C/A	0.27	0.99	1.04	0.58	0.14	0.91	0.96	0.54	0.27	0.70	0.98	0.73	0.13	0.6779	0.0001 ^	0.0769
**Metabolism**																
CO_2_%	1.32 ^a^	6.51 ^b^	14.01 ^cde^	12.26 ^bcd^	2.87 ^a^	11.86 ^cd^	20.36 ^ef^	14.58 ^d^	3.63 ^a^	8.55 ^bc^	22.49 ^f^	20.43 ^e^	1.54	0.0001 +	0.0001 +^	0.0016 *
ASP% ^c^	28.48 ^d^	11.28 ^a^	18.58 ^c^	36.16 ^f^	34.56 ^ef^	12.34 ^ab^	28.47 ^d^	29.64 ^de^	33.53 ^def^	16.53 ^bc^	35.88 ^f^	31.51 ^def^	1.87	0.0001 +	0.0001 +^	0.0001 *
ESP% ^c^	70.19 ^ef^	80.33 ^g^	65.08 ^de^	51.58 ^bc^	62.57 ^d^	75.14 ^f^	50.15 ^bc^	55.78 ^c^	62.85 ^d^	73.90 ^f^	38.28 ^a^	48.05 ^b^	2.29	0.0001 +	0.0001 +^	0.0001 *
O/M	0.30 ^b^	0.20 ^a^	0.35 ^b^	0.48 ^cd^	0.37 ^b^	0.25 ^ab^	0.50 ^d^	0.44 ^c^	0.37 ^b^	0.26 ^ab^	0.62 ^e^	0.52 ^d^	0.02	0.0001 +	0.0001 +^	0.0001 *

Data are least square means and pooled standard error means (SEM). ^abcdefg^ Data with different letter differ (*p* < 0.05). Con, Control sows, Clof 0.25, sows received 0.25% of dietary clofibrate and Clof 0.5, sows received 0.5% dietary clofibrate. ASP, acid soluble products and ESP, esterified products. C/A, CO_2_/ASP and O/M, oxidation/metabolism. * Indicating the difference between clofibrate and control from the analysis of contrast. + linear response. ^ quadratic response.

**Table 3:** Effect of maternal clofibrate and postnatal age on intestinal mucosa gene expressions of piglets during neonatal suckling period.

	Clofibrate (%)					Age (Day)						Clof *Age
Genes	Con	Clof 0.25	Clof 0.50	SEM	*p*-Value	D1	D7	D14	D19	SEM	*p*-Value	*p*-Value
	Fold					Fold						
**INTESTINE**												
*ACOX1*	2.75	2.60	2.34	0.24	0.423	2.68	2.46	2.63	2.50	0.28	0.915	0.037 *
*CPT1A*	1.01	1.04	1.19	0.19	0.737	1.48 ^b^	1.09 ^b^	0.58 ^a^	1.16 ^b^	0.22	0.046 ^	0.543
*CPT1B*	1.14	0.98	1.18	0.21	0.772	1.39	1.35	0.65	1.02	0.25	0.161	0.177
*FABP2*	0.82	1.17	1.04	0.13	0.171 ^#^	0.56 ^a^	0.79 ^a^	1.25 ^b^	1.43 ^b^	0.15	0.001 ^+^	0.400
*PPARα*	1.23	0.89	1.10	0.15	0.266	1.28 ^b^	1.41 ^b^	0.96 ^ab^	0.66 ^a^	0.18	0.019 ^+^	0.106
*RXRα*	1.15	0.66	0.95	0.17	0.123 ^#^	1.22 ^bc^	1.34 ^c^	0.79 ^ab^	0.33 ^a^	0.20	0.003 ^+^	0.244
***CPT1A*/*CPT1B***												
Ratio	1.04	1.01	0.93	0.09	0.662	1.00 ^ab^	0.89 ^a^	0.84 ^a^	1.25 ^b^	0.09	0.034 ^	0.087 *

Data are least square means and pooled standard error means (SEM). ^abc^ Data with different letter differ (*p* < 0.05). Con, Control sows, Clof 0.25, sows received 0.25% of dietary clofibrate and Clof 0.5, sows received 0.5% dietary clofibrate. Genes: *ACOX,* peroxisomal acyl-CoA oxidase; *CPT1A*, carnitine palmitoyltransferase 1A, *CPT1B*; carnitine palmitoyltransferase 1B; *FABP2*, fatty acid binding protein 2; *PPARα*, peroxisome proliferator-activated receptor alpha; *RXRα*, retinoid X receptor alpha. ^#^ Contrast control vs. clofibrate for *FABP2* (*p* = 0.078); for *RXRα* (*p* = 0.095); ^+^ linear response (*p* < 0.05) and ^ quadratic response (*p* < 0.05). * Significant interaction was observed for *ACOX*1 in which supplementation of clofibrate increased its expression on d1 but not after d1. The ratio of *CPT1A/CPT1B* decreased on d1.

## Data Availability

Data is contained within the article and will be available in PubAg.

## Supplementary Materials

The following supporting information can be downloaded at: https://www.mdpi.com/article/10.3390/ijms26178691/s1.

## Abbreviations

The following abbreviations are used in this manuscript:

ACOX1	Acyl-CoA oxidase 1
ASP	Acid soluble products
CPT	Carnitine palmitoyltransferase
ESP	Esterified products
FA	Fatty acid
FABP2	FA-binding protein 2
HMGCS	3-hydroxy-3-methylglutaryl-CoA synthase
NEFA	Non-Esterified Fatty Acids
PPARα	Peroxisome proliferator-activated receptor alpha
RXRα	Retinoid X receptor alpha
TCA	Tricarboxylic acid cycle
TG	Triglycerides
