# Supplementary material for: Effects of Maternal Clofibrate Supplementation During Gestation and Lactation on Intestinal Fatty Acid Oxidation of Suckling Piglets
Source: Int J Mol Sci. 2025 Sep 6;26(17):8691. doi: 10.3390/ijms26178691 (PMC12429302; doi:10.3390/ijms26178691)
Supplement: Supplementary file 1 [file ijms-26-08691-s001.zip › ijms-3748483-supplementary.pdf]

Supplemental Table S1. Primers used in this study with expected amplicon size post RT-qPCR.

	Forward primer (5'-3'), Sen	Reverse primer (5'-3'), Anti	Amplicon Size, bp	NCBI (Gene Bank)*
ACOX1	GGTCCATCCACGCTGTCTTA	CACGTGGGTGACTTGAGACT	119	NM_001101028.1
FABP2	CAACGAGTGGATAATGGAAAAGAGT	CCTCTTGGCTTCTACTCCTTCA	99	NM_001031780.1
CPT1A	GCTGACGATGGTTATGGGGT	TCCCGAAGCGATGAGAATCC	109	NM_001129805.1
CPT1B	GCCTGACCTATGAAGCCTCG	TGAACGAAGGCTGTGGACTC	94	NM_001007191.1
PPARα	GCTGGACGACAGTGACCTTT	AGCACATGCACGATACCCTC	117	NM_001044526.1
RXRα	GTCCTCTTCAACCCGGACTC	CTGCTCGGGGTACTTGTGTT	114	DQ279926.1
RPL9	GCAACTGTTCGCACCATCTG	CGACGTTGATGGGGAAGTGA	109	NM_001243481.1

*Gene Bank numbers provided to demonstrate the genes of the pig genome referenced.
